# Molecular mechanisms, physiological roles, and therapeutic implications of ion fluxes in bone cells: Emphasis on the cation‐Cl^−^ cotransporters

**DOI:** 10.1002/jcp.30879

**Published:** 2022-09-20

**Authors:** Alexandre P. Garneau, Samira Slimani, Ludwig Haydock, Thy‐René Nsimba‐Batomene, Florence C.M. Préfontaine, Mathilde M. Lavoie, Laurence E. Tremblay, Marie‐Jeanne Fiola, Fabrice Mac‐Way, Paul Isenring

**Affiliations:** ^1^ Department of Medicine, Nephrology Research Group Laval University Québec Québec Canada; ^2^ Service de Néphrologie—Transplantation Rénale Adultes, Hôpital Necker‑Enfants Malades, AP‑HP, Inserm U1151 Université Paris Cité, rue de Sèvres Paris France

**Keywords:** bone disorders, K^+^‐Cl^−^ cotransporters, Na^+^‐Cl^−^ cotransporters, Na^+^‐K^+^‐Cl^−^ cotransporters, osteoblasts, osteoclasts

## Abstract

Bone turnover diseases are exceptionally prevalent in human and come with a high burden on physical health. While these diseases are associated with a variety of risk factors and causes, they are all characterized by common denominators, that is, abnormalities in the function or number of osteoblasts, osteoclasts, and/or osteocytes. As such, much effort has been deployed in the recent years to understand the signaling mechanisms of bone cell proliferation and differentiation with the objectives of exploiting the intermediates involved as therapeutic preys. Ion transport systems at the external and in the intracellular membranes of osteoblasts and osteoclasts also play an important role in bone turnover by coordinating the movement of Ca^2+^, PO_4_^2−^, and H^+^ ions in and out of the osseous matrix. Even if they sustain the terminal steps of osteoformation and osteoresorption, they have been the object of very little attention in the last several years. Members of the cation‐Cl^−^ cotransporter (CCC) family are among the systems at work as they are expressed in bone cells, are known to affect the activity of Ca^2+^‐, PO_4_^2−^‐, and H^+^‐dependent transport systems and have been linked to bone mass density variation in human. In this review, the roles played by the CCCs in bone remodeling will be discussed in light of recent developments and their potential relevance in the treatment of skeletal disorders.

## Graphical abstract

Ion transport systems in the membranes of osteoblasts and osteoclasts play a key role in bone remodeling by coordinating the movement of Ca^2+^, PO_4_^2−^, and H^+^ ions in and out of the osseous matrix. Based on the evidence available, the cation‐Cl^−^ cotransporters could act as unexpectedly important players in this regard. Here, we explain more specifically why by revisiting the data published on the subject and the mechanisms of mineral flux during bone remodeling.

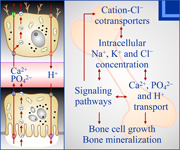

## 1 INTRODUCTION

Osteoporosis is the most common form of bone turnover disease (BTD). It affects ∼10% of the population after the age of 50 and increases mortality by ∼10% when it leads to hip fractures (Rosen, 2000). Another form of BTD is known as osteopetrosis but is far less common. Although bones are denser than normal in this disease, they are still prone to fractures (Cleiren et al., 2001; Josephsen et al., 2009; Kornak et al., 2001; Margolis et al., 2008). Regardless of their types or etiologies, BTDs are all characterized by abnormalities in the function and/or number of osteoblasts, osteoclasts, and/or osteocytes (Rosen, 2000). As such, the molecular players of bone cell proliferation and differentiation have been the object of great attention over the last decade.

Among the players of interest are the estrogen receptor complexes (Pickar et al., 2018) and members of the RANKL/NFκB/NFAT and Wnt/β‐catenin/Runx2 signaling pathways (Baron & Gori, 2018; Lacey et al., 2012). A few of them have even become targets for the treatment of BTDs (Lacey et al., 2012). Ion transport systems are also expressed in bone cells to ensure the flux of Ca^2+^, PO_4_^2−^, and H^+^ ions in and out of the bone matrix and have been linked to many types of bone disorders in animals (Table 1). Somewhat surprisingly, however, they have been the object of very little interest during the last several years.

**Table 1:** Bone turnover disorders linked top ion transport systems in human and animal models

Gene	Transmission	Manifestations	OMIM	References
(A) Human				
*ATP6V0A3*	Recessive	Recessive osteopetrosis 1^a^	Gene‐604592	Kornak et al. (2000)
*CA2*	Recessive	Recessive osteopetrosis 2^b^	Gene‐611492	Sly et al. (1985)
*CLCN5*	X‐linked	Hypophosphatemic rickets 1^c^	Gene‐300008	Fisher et al. (1994)
*CLCN7*	Dominant	Dominant osteopetrosis 2^a^	Gene‐602727	(4) Cleiren et al. (2001)
*CLCN7*	Recessive	Recessive osteopetrosis 4	Gene‐602727	(4) Cleiren et al. (2001)
*SLC12A3*	Recessive	Gitelman syndrome, ↑ BMD	Gene‐600968	Nicolet‐Barousse et al. (2005)
*SLC9A3R1*	Dominant	Hypophosphatemic osteoporosis 2	Gene‐604990	Karim et al. (2008)
*SLC34A1*	Dominant	Hypophosphatemic rickets 1, osteomalacia	Gene‐182309	Prié et al. (2002)
*SLC34A3*	Recessive	Hypercalciuric hypophosphatemic rickets	Gene‐609826	Bergwitz et al. (2006)

**Gene**	**Genotype**	**Phenotype**	**Background**	**References**
(B) Mouse				
*ATP6V0A3*	−/+	Osteopetrosis (similar to human)	C57BL/6J	Ochotny et al. (2011)
*CA2*	−/−	Osteopetrosis (similar to human)	C57BL/6J	Margolis et al. (2008)
*PMCA1*	−/+	Osteopenia, ↑ *n* osteoclasts	129 × 1/SvJ	Kim et al. (2012)
*PMCA4*	−/−	Osteopenia, ↑ *n* osteoclasts	129 × 1/SvJ	Kim et al. (2012)
*CLCN3*	−/−	Low osteoclastic resorption activity in vitro	ICR	Okamoto et al. (2008)
*CLCN5*	−/−	↑ General bone turnover	C57BL × 129SV	Silva et al. (2003)
*CLCN7*	−/−	Osteopetrosis (similar to human)	C57BL/6 × 129SV	Kornak et al. (2001)
*NCC*	−/−	↑ Osteoblastic differentiation, ↑ BMD	C57BL/6	Nicolet‐Barousse et al. (2005)
*SLC34A1*	−/−	Hypophosphatemic rickets (similar to human)	C57BL/6 × 129SV	Beck et al. (1998)
*SLC34A2*	−/−	↓ BMD, ↑ *n* osteoclasts under low‐phosphorus diet	C57BL/6 × 129SV	Knöpfel et al. (2017)
*SLC4A2*	−/−	Osteopetrosis with ballooned osteoclasts	129S6/SvEv	Josephsen et al. (2009)
*TRPV5*	−/−	↓ Bone mass and mineralization	C57BL/6 × 129SV	van der Eerden et al. (2016)
*TRPV6*	−/−	Osteopenia, ↑ osteoclastic resorption activity	C57BL/6J	F. Chen et al. (2014)

*Note*: Some of the ion transport systems listed could be linked to the manifestations specified through renal calcium or phosphate wasting but were still included given that they are expressed in bone cells. (A) Human. Source of data was OMIM.org database. (B) Mouse. Examples of the background used for gene inactivation are provided. ↑, increase; ↓, decrease; BMD, bone mineral density; *n*, number.

^a^ Albers‐Schonberg disease.

^b^ Guibaud‐Vainsel syndrome.

^c^ Dent disease.

In this review, the importance and role of ion transport systems in BTDs will be revisited in light of new developments and hypothetical perspectives. The cation‐Cl^−^ cotransporter (CCC) family will be paid special consideration given that six of its members have been detected in the skeleton and that one of them has been found to affect bone mineral density in human (Cheng et al., 2018; Nicolet‐Barousse et al., 2005; Wasnich et al., 1983). For these reasons, and because their pharmacological inhibition is well‐tolerated, a relevant question that needs to be addressed is whether the CCCs are skeletal targets of underestimated clinical potential (Garneau & Isenring, 2019; Garneau et al., 2017; Garneau, Marcoux et al., 2019; Garneau, Slimani et al., 2019; Garneau et al., 2020; Marcoux et al., 2017).

## 2 WHAT ARE THE CCCS?

### 2.1 Overview

The CCCs are a group of cell surface membrane proteins that are highly homologous to each other and ubiquitously distributed (Garneau & Isenring, 2019; Garneau et al., 2017; Garneau, Marcoux et al., 2019; Garneau, Slimani et al., 2019; Garneau et al., 2020; Marcoux et al., 2017). They fall into four phylogenetic clades as follows: (1) the Na^+^‐dependent cotransporters, that is, Na^+^‐K^+^‐Cl^−^ cotransporter 1 (NKCC1; SLC12A2), NKCC2 (SLC12A1), and Na^+^‐Cl^−^ cotransporter (NCC; SLC12A3), (2) the Na^+^‐independent cotransporters, that is, K^+^‐Cl^−^ cotransporter 1 (KCC1; SLC12A4), KCC2 (SLC12A5), KCC3 (SLC12A6), and KCC4 (SLC12A7), (3) CCC8 (SLC12A9), and (4) CCC9 (SLC12A8).

The Na^+^‐dependent CCCs are known to mediate the electroneutral cotranslocation of Cl^−^ and Na^+^ or K^+^ into cells (NKCC1, NKCC2, and NCC), and the Na^+^‐independent CCCs, that of Cl^−^ and K^+^ out of cells (KCCs). In doing so, they affect the activity of coexpressed ion transport systems including Na^+^‐, K^+^‐, and Cl^−^‐dependent Ca^2+^, PO_4_^2−^, and H^+^ transporters as well as Na^+^, K^+^, Cl^−^, and water channels (Garneau & Isenring, 2019; Garneau et al., 2017; Garneau, Marcoux et al., 2019; Garneau, Slimani et al., 2019; Garneau et al., 2020; Marcoux et al., 2017). As such, they also affect the global ion concentration, membrane potential, and volume of cells. In fact, they exert several of their roles by acting on these coexpressed ion transport systems (Garneau & Isenring, 2019; Garneau et al., 2017; Garneau, Marcoux et al., 2019; Garneau, Slimani et al., 2019; Garneau et al., 2020; Marcoux et al., 2017; Markadieu & Delpire, 2014; Yamamoto et al., 2002). As for CCC8 and CCC9, they have still not been ascribed a precise transport function as of yet (Caron et al., 2000; Daigle et al., 2009).

For the Na^+^‐dependent CCCs, ion cotransport is stimulated by phosphorylation of the N‐terminus, and for the Na^+^‐independent CCCs, it is stimulated by dephosphorylation of the C‐terminus (Darman & Forbush, 2002; Gimenez & Forbush, 2003; Pacheco‐Alvarez et al., 2006; Rinehart et al., 2009). The regulatory factors that act on these domains include members of the WNK kinases/SPAK‐OSR1 signaling pathway (Mercier‐Zuber & O'Shaughnessy, 2011; Piechotta et al., 2002) and a number of accessory proteins (Boyden et al., 2012; Liedtke et al., 2003; Ponce‐Coria et al., 2012; Reiche et al., 2010; Simard et al., 2004; Smith et al., 2013). They probably underlie the effects of many environmental cues, hormones, or peptides on carrier activity (Gimenez & Forbush, 2003; Marcoux et al., 2017, 2019; Sandberg et al., 2007).

The ion‐transporting CCCs are all inhibited by loop and/or thiazide diuretics but to various degrees. However, these compounds reach much higher concentrations in the lumen of renal tubules than in other compartments or tissues such that they cannot inhibit the extra‐renal CCCs potently without inducing a substantial natriuretic response (Ponto & Schoenwald, 1990). CCC‐interacting compounds that have limited access to the tubular ultrafiltrate (Ishizaki et al., 2009) could thus reveal beneficial in the treatment of miscellaneous disorders where excessive cation‐Cl^−^ cotransport is believed to play an important pathophysiological role (H. Chen et al., 2005; Dzhala et al., 2005; Oppermann et al., 2006; Solymosi et al., 2013; Steffensen et al., 2018; Weidenfeld & Kuebler, 2017).

### 2.2 Membrane potential and CCC

If the CCCs can alter membrane potential while they are not primarily electrogenic, it is because they harbor transport sites for at least one anion and one cation while the baseline anion‐to‐cation conductance ratio of cell membranes is rarely equal to 1.0. For instance, this ratio is higher for Cl^−^ than it is for K^+^ in many types of neurons such that K‐Cl influx by a CCC exerts a depolarizing effect, and K‐Cl efflux, a hyperpolarizing effect (Delpire & Gagnon, 2018). When the anion‐to‐cation conductance ratio is higher for K^+^, such as in GABA_A_‐expressing vestibular and inner ear spiral ganglia (Markadieu & Delpire, 2014; Yamamoto et al., 2002), K‐Cl influx by a CCC exerts a hyperpolarizing effect, and K‐Cl efflux, a depolarizing effect.

There is evidence to suggest that K^+^ channel activity at the surface of bone cells is an important determinant of membrane conductance (Chow et al., 1984; Edelman et al., 1986; Gu et al., 2001; Kelly et al., 1992; Ravesloot et al., 1990; Sims et al., 1991; Wilson et al., 2011). It could thus play the key role of orienting the movement of many ions by the electrogenic transport pathways of both osteoclasts and osteoblasts in response to a change in CCC activity. As will be discussed later, this possibility is supported further by the opposed repercussions of NCC and NKCC inhibition on bone cell signaling and long‐term mineral density. It will be used as a working model to propose an integrated portrait of ion fluxes by the skeletal *transportome*.

## 3 ION TRANSPORT IN BONE CELLS

### 3.1 Osteoclasts

**Figure 1:**
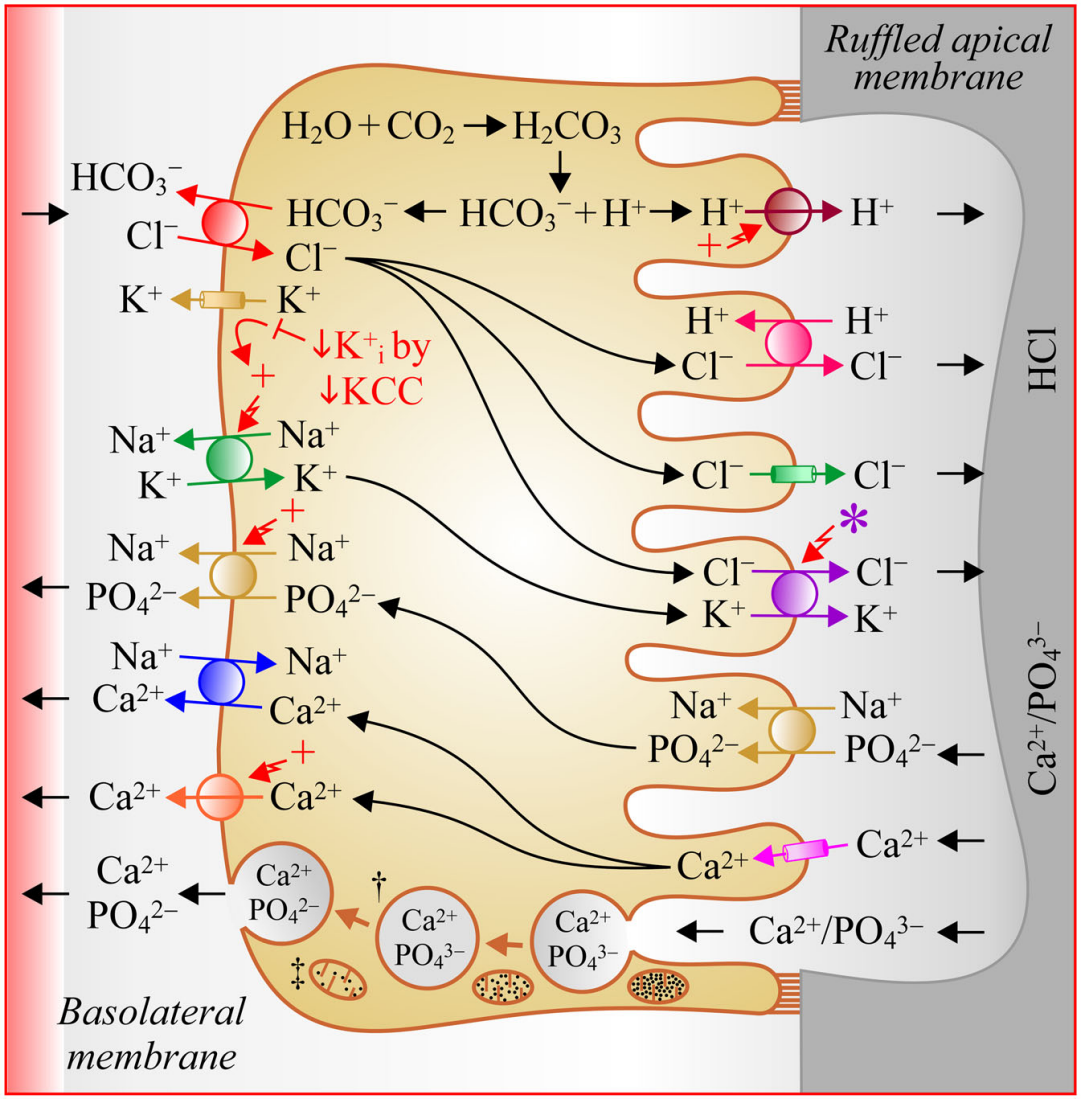
Model of ion transport in osteoclasts and contribution of KCC1. Serosal ion transport systems (from top to bottom): 1Cl^−^/1HCO_3_^−^ exchanger AE2/SLC4A2 (Wu et al., 2008); K^+^ channels (many types); Na^+^/K^+^‐ATPase α1β2 (Francis et al., 2002; Makihira et al., 2011); 3Na^+^−1PO_4_^2−^ cotransporter NaPi2a/SLC34A1 (Albano et al., 2015; Khadeer et al., 2003); 3Na^+^/1Ca^2+^ exchanger NCX1 (Moonga et al., 2001); Ca^2+^‐ATPase PMCA1 (Bekker & Gay, 1990; Kim et al., 2012). Apical ion transport systems (from top to bottom): vacuolar H^+^‐ATPase ATP6V0a3d2V1B2C1 (Feng et al., 2009); 1H^+^/2Cl^−^ exchanger CLCN7 (Kornak et al., 2001); Cl^−^ channel; KCC1/SLC12A4 and KCC2/SLC12A5 (Kajiya et al., 2006); 1Na^+^−2PO_4_^2−^ cotransporters PIT1/SLC20A1 and PIT2/SLC20A2^4^ (Gupta et al., 1996); Ca^2+^ channel TRPV5 (van der Eerden et al., 2005; Yan et al., 2011). Signs used: *, CCC; ^†^, transcytotic vesicles; ^‡,^ Ca^2+^‐rich mitochondria; +, depolarization; sign –, hyperpolarization; straight arrow, leads to; broken arrow, activates; and T sign, inhibits. Note that the subcellular localization and nature of the Ca^2+^ and PO_4_^2−^ transport systems are still incompletely established. CCC, cation‐Cl^−^ cotransporter.

Osteoclasts play a key role in the formation of resorptive pits by secreting H^+^ through their ruffled apical border along with Cl^−^ to neutralize the proton charge. As described in Figure 1 (see top half) and accompanying legend, the ion transport systems at play are a vacuolar H^+^‐ATPase (Feng et al., 2009) and a H^+^/2Cl^−^ exchanger (Kornak et al., 2001). Ion transport systems of importance for the formation of resorptive pits are also present on the serosal side (Francis et al., 2002; Makihira et al., 2011; Wu et al., 2008). They include a Cl^−^/HCO_3_^−^ exchanger that allows for higher Cl^−^_i_‐to‐Cl^−^_o_ ^1^ concentration gradients to drive H^+^/2Cl^−^ exchange and for higher HCO_3_^−^ efflux to drive the cellular synthesis of H^+^.

A few years ago, RT‐PCR analyses and protein expression studies led Kajiya et al. (2006) to find that KCC1 and KCC2 were both expressed in primary cultures of mouse osteoclasts while KCC3 and KCC4 were both absent. However, there are several transcripts for KCC3 in human bone RNA databanks. As such, this other isoform could be expressed in osteoblasts and/or osteocytes more specifically or more abundantly.

In their study, Kajiya et al. (2006) also observed that pit formation in calcified dentine slices added with mouse osteoclasts in culture was suppressed by KCC1 antisense oligonucleotides and that Cl^−^_i_ and H^+^_i_ in these cells were both increased through pharmacological inhibition of the KCCs. As illustrated through Figure 1 (see middle half), it was thus proposed that the role of KCC1 in osteoclasts was to hamper the transfer of H^+^ from pit to cytosol by providing an added extrusion mechanism for Cl^−^. In this respect, loss‐of‐function mutations in the H^+^/2Cl^−^ exchanger CLCN7 (Table 1) have been found to cause osteopetrosis in both mouse models and human (Cleiren et al., 2001; Kornak et al., 2001).

The distribution of KCC1 in osteoclasts has still not been clearly established. In either membrane, the carrier would still be expected to sustain Cl^−^/HCO_3_^−^ exchange (and H^+^ synthesis) by decreasing Cl^−^_i_ and to sustain Na^+^/K^+^‐ATPase activity by decreasing K^+^_i_. If, as hypothesized and indicated in Figure 1 (see middle half), it led outward K^+^ conductance to be lower as well, it would increase net Na^+^/K^+^‐ATPase and H^+^‐ATPase activity further. Were KCC1 localized on the ruffled side more specifically, it would also allow for an added Na^+^/K^+^‐ATPase‐driven route for Cl^−^ secretion to sustain pit acidification.

All of the KCCs can translocate NH_4_^+^ through their K^+^‐transport site (Bergeron et al., 2003) and could thus affect the pH of osteoclasts in doing so. NH_4_^+^‐Cl^−^ cotransport by these carriers would be in fact inwardly directed in that the NH_4_^+^_o_‐to‐NH_4_^+^_i_ gradient is above 2.0 in most cell types (Evans & Turner, 1998). Interestingly, the KCCs have also been shown to have similar apparent affinities for NH_4_^+^ and K^+^ based on in vitro studies (Bergeron et al., 2003), implying that they could allow for substantial NH_4_^+^ uptake in H^+^‐ATPase expressing cells. If it were localized on the basolateral side, KCC1 would also provide the vacuolar pump with an additional source of substrate on the apical side.

During bone resorption, Ca^2+^ and PO_4_^2−^ are released from the matrix and returned to the circulation. As described in Figure 1 (bottom half) and accompanying legend, these ions are transported from the apical to basolateral side of osteoclasts through transcytotic vesicles (along with digested bone matrix) and mitochondria^2^ (Kawahara et al., 2009; Zhao, 2012). As shown again in Figure 1 (bottom half), the presence of transport systems for Ca^2+^ and PO_4_^2−^ at the surface (Albano et al., 2015; Bekker & Gay, 1990; van der Eerden et al., 2005; Gupta et al., 1996; Khadeer et al., 2003; Kim et al., 2012; Moonga et al., 2001; Yan et al., 2011) of both membranes suggests that these ions are also recycled through protein‐facilitated transepithelial routes and that their movements should thus be affected by the activity of KCC1 and/or other CCCs.

There is growing evidence to suggest that ion transport systems do more than merely affect the activity of each other, but that they also affect the activity of signaling intermediates. In osteoclasts, such intermediates—those of the RANKL/NFκB/NFAT pathway in particular (Grossinger et al., 2018; Heeschen et al., 2002; Schwab et al., 2012)—must in turn act on many of the expressed ion transport systems for functional H^+^‐ and Cl^−^‐secreting or Ca^2+^‐ and PO_4_^2−^‐absorbing cell units to be formed. Evidence in support of this contention is that the H^+^/2Cl^−^ exchanger CLCN7 is now known a target gene of NFAT along with cathepsin K and TRAP (Park et al., 2017; Sasaki et al., 2009).

The functional relevance of K^+^‐Cl^−^ cotransport in osteoclasts could have been assessed more readily by characterizing the available mouse models or the known human disorders of KCC inactivation or overactivation (Howard et al., 2002; Rust et al., 2007) through relevant phenotyping studies. As it stands, however, there are no findings reported in the literature on this matter. Bone‐specific conditional KCC mouse models do not appear to be available either but would allow determining whether K^+^‐Cl^−^ cotransport in osteoclasts affects bone resorption directly or systemically.

### 3.2 Osteoblasts

**Figure 2:**
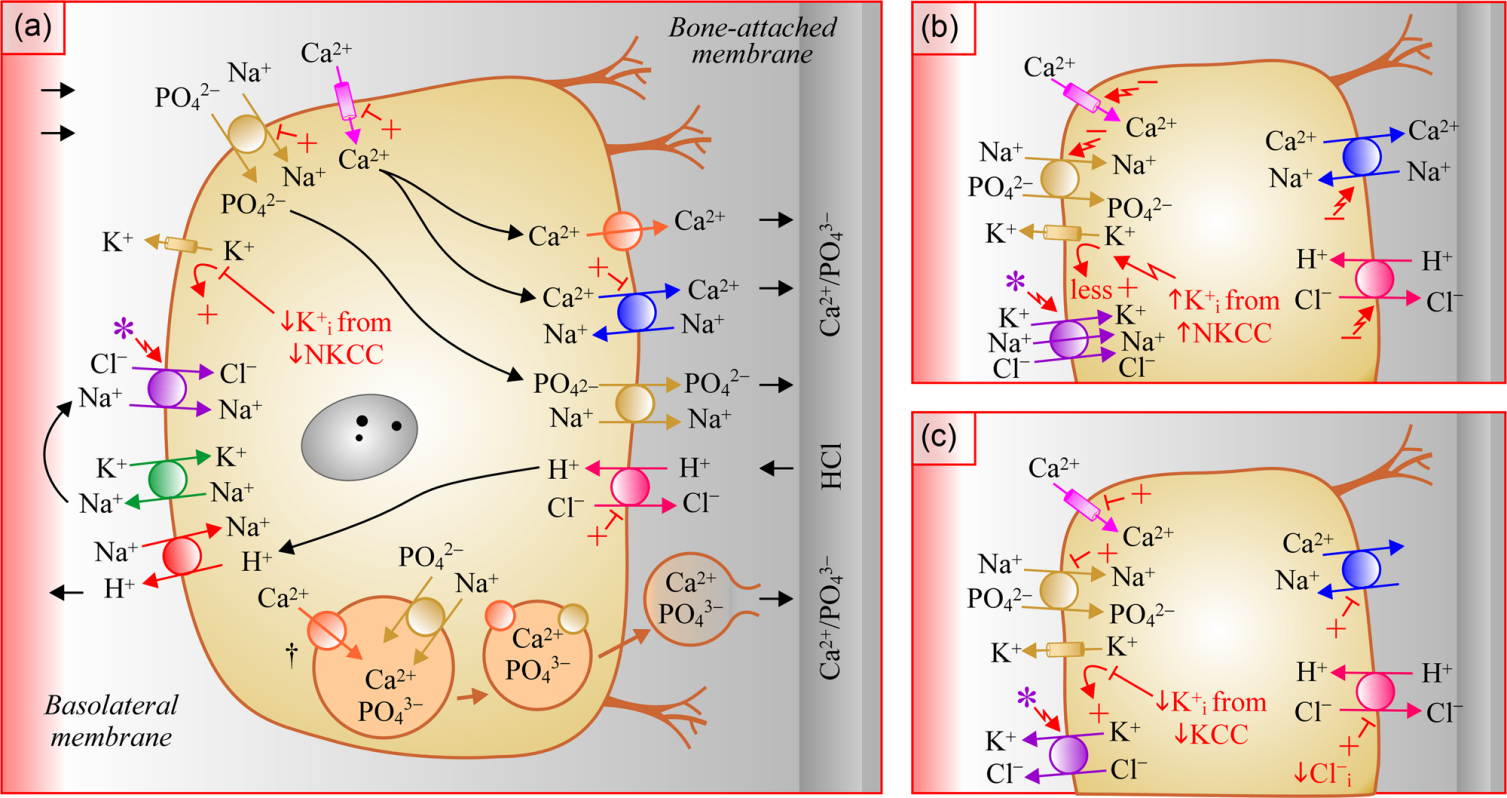
Model of ion transport in osteoblasts and contribution of different CCCs. (a) Contribution of NCC. Serosal ion transport systems (from top to bottom): Ca^2+^ channel TRPV5 and/or TRPV6 (F. Chen et al., 2014; Little et al., 2011; Wade‐Gueye et al., 2012; Weber et al., 2001); 3Na^+^−1PO_4_^2−^ cotransporters NaPi2a/SLC34A1 and NaPi2b/SLC34A2 (Lundquist, 2002; Wang et al., 2013); K^+^ channel (many types); NCC/SLC12A3 (Dvorak et al., 2007); Na^+^/K^+^‐ATPases α1β1 and α1β2 (Francis et al., 2002); 1Na^+^/1H^+^ exchangers NHE1/SLC9A1 and NHE6/SLC9A6 (L. Liu et al., 2011). Apical transport systems (from top to bottom): Ca^2+^‐ATPase PMC1, PMCA1b, and PMCA2 (Francis et al., 2002; Meszaros & Karin, 1993); 3Na^+^/1Ca^2+^ exchangers NCX3/SLC8A3>NCX1/SLC8A1 (Lundquist et al., 2000; Sosnoski & Gay, 2008; Stains et al., 2002); 2Na^+^−2PO_4_^2−^ cotransporters PIT1/SLC20A1 and PIT2/SLC20A2^4^ (Beck‐Cormier et al., 2019); 1H^+^/2Cl^−^ exchangers CLCN3 and CLCN5 (Larrouture et al., 2015). Matrix vesicle ion transport systems: 2Na^+^−1PO_4_^2−^ cotransporters PIT1/SLC20A1 and PIT2/SLC20A2^5^ (Nielsen et al., 2001; Suzuki et al., 2006); Ca^2+^‐ATPase ANXA1, ANXA2, ANXA5, and ANXA6^5^ (Balcerzak et al., 2008; Kirsch et al., 1997; Kirsch, 2005; Z. Xiao et al., 2007). (b) Contribution of NKCC1. Serosal ion transport systems: NKCC1/SLC12A2 in panel C2 (Lee et al., 2003); others same as in panel (a). Apical transport systems: same as in panel (a). (c) Contribution of NKCC. Serosal ion transport systems: KCC1/SLC12A4, KCC2/SLC12A5, KCC3/SLC12A6, and/or KCC4/SLC12A7 (Brauer et al., 2003); others same as in panel (a). Apical transport systems: same as in panel (a). Signs used in panels (a), (b), and (c) are as in legend to Figure 1 except for ^†^ that points to matrix vesicles. Note that the subcellular localization and nature of the Ca^2+^ and PO_4_^2−^ transport systems are still incompletely established. CCC, cation‐Cl^−^ cotransporter.

An essential step in osteoformation is the skeletal uptake of Ca^2+^ and PO_4_^2−^ from the circulation (see Figure 2a and legend). This uptake is achieved by Ca^2+^ channels (F. Chen et al., 2014; Little et al., 2011; Wade‐Gueye et al., 2012; Weber et al., 2001) and Na^+^‐PO_4_^2−^ cotransporters (Lundquist, 2002; Wang et al., 2013) at the basolateral membrane of osteoblasts with the aid of Na^+^/K^+^‐ATPases to ensure a favorable (inside negative) gradient for the movement of Ca^2+^ and PO_4_^2−^ ions and a favorable Na^+^_o_‐to‐Na^+^_i_ gradient for the movement of PO_4_^2−^ ions (Francis et al., 2002). A large fraction of the absorbed Ca^2+^ and PO_4_^2−^ ions is also taken up by intracellular matrix vesicles from the cytosol (see bottom of Figure 2a and legend) through additional Na^+^‐PO_4_^2−^ cotransporters (Nielsen et al., 2001; Suzuki et al., 2006) and Ca^2+^‐ATPases in the membrane of these organelles (Balcerzak et al., 2008; Kirsch et al., 1997; Kirsch, 2005; Z. Xiao et al., 2007).

Another essential step in osteoformation is the transfer of Ca^2+^ and PO_4_^2−^ ions from osteoblasts to osteoid bone (see Figure 2a and legend). It is achieved mainly through the apical secretion of the matrix vesicles themselves that are freed of their content into this space (Anderson et al., 2005; Hasegawa et al., 2017; Zhao, 2012). Some level of secretion also occurs via a Ca^2+^‐ATPase (Meszaros & Karin, 1993), Na^+^/Ca^2^^+^ exchangers (Lundquist et al., 2000; Sosnoski & Gay, 2008; Stains et al., 2002), and Na^+^‐PO_4_^2−^ cotransporters (Beck‐Cormier et al., 2019) through the bone attached membrane domain. Of notice, demineralization of forming bone is prevented in this setting by the transcellular reabsorption of protons via apical H^+^/2Cl^−^ exchangers (Larrouture et al., 2015) and serosal Na^+^/H^+^ exchangers (L. Liu et al., 2011).

As will be seen below, NCC and NKCC1 are both expressed at the surface of osteoblasts and are thus likely to affect the activity of coexpressed Ca^2+^, PO_4_^2−^, and H^+^ transport systems. However, they are not predicted to do so analogously based on their effect on K^+^ conductance given that one is K^+^‐dependent while the other K^+^‐independent (compared Figure 2a with 2b). The same could be said of the KCCs compared to NKCC1 given that K^+^ movement by these carriers is in the opposite direction (compare Figure 2b with 2c).

Among the various ion‐transporting CCCs, NCC is the isoform that has drawn the most interest in the field of BTD. Inter alia, its inactivation in human through long‐term administration of thiazides or homozygous loss‐of‐function mutations has been found to prevent bone mass loss in a number of observational studies. A meta‐analysis by Cheng et al. (2018) has recently confirmed that thiazides could be beneficial in the treatment of osteoporosis but also led to the conclusion that higher‐quality studies were required to obtain stronger evidence to this effect.

Thiazides have been said to preserve bone mass because of their positive impact on Ca^2+^ homeostasis, that is, because NCC inhibition in the renal and intestinal epithelia causes these cell linings to exhibit higher levels apical Ca^2+^ conductance and basolateral Na^+^/Ca^2+^ exchange (Alexander & Dimke, 2017; Cheng et al., 2018; Hsu et al., 2015; Nicolet‐Barousse et al., 2005). Yet, there is also evidence to suggest that thiazides could preserve bone mass by acting on the skeleton directly (Dvorak et al., 2007; Hsu et al., 2015; Nicolet‐Barousse et al., 2005). In particular, a study by Dvorak et al. (2007) has shown that inactivation of Na^+^‐Cl^−^ cotransport in cultured osteoblasts led to increased cell differentiation and nodule formation.

A direct effect of thiazides on osteoblastogenesis would suggest more specifically that it is relayed through the involvement of differentiating factors (such as those of the Wnt/β‐catenin/Runx2 pathway for instance) and that the activity, expression or distribution of such factors would thus be sensitive to changes in intracellular ion concentration or cell volume. In this regard, interestingly, human brain vascular smooth myocytes are prevented from proliferating and undergoing Wnt/β‐catenin/Runx2 activation in parallel when their H^+^/2Cl^−^ exchanger CLCN2 is inhibited pharmacologically (Lu et al., 2018).

**Figure 3:**
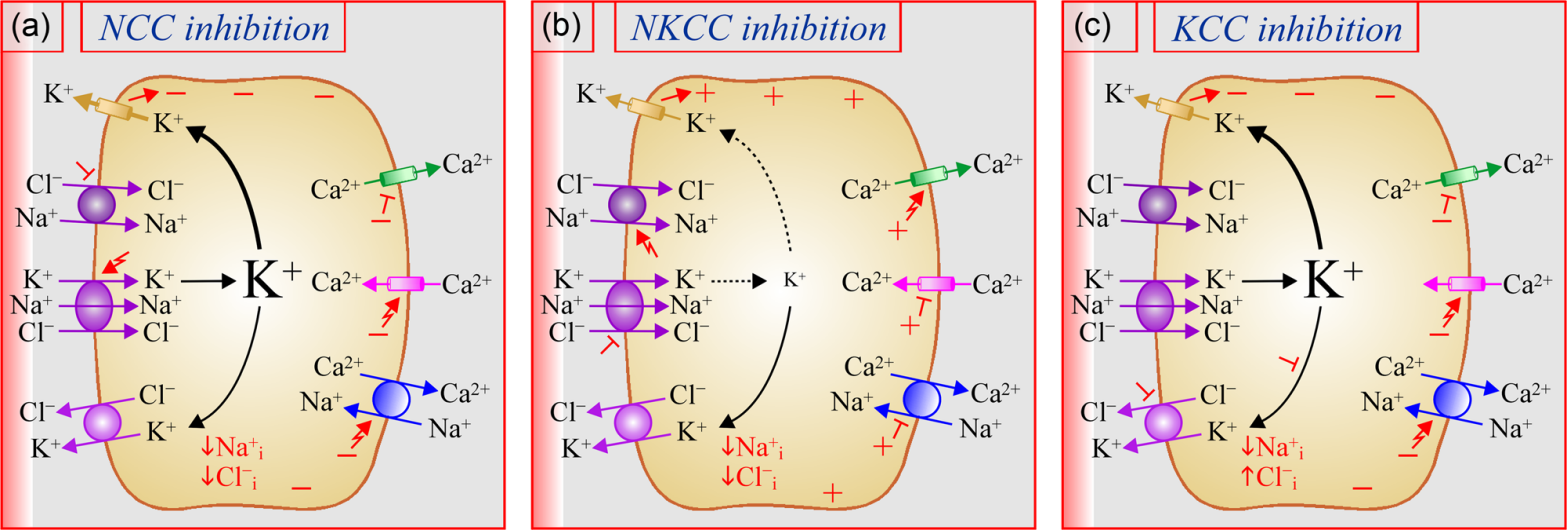
Presumed effect of CCC inhibition on membrane potential in bone cells. The basolateral membranes of osteoclasts and osteoblasts are presumed to be more conductive to cations than anions under normal circumstances due to the presence of several types of K^+^ channels including Kir2.1, Kv1.3, IK_Ca_, SK_Ca_, BK_Ca,_ and a number of P2X to name a few (Edelman et al., 1986; Gu et al., 2001; Kelly et al., 1992; Ravesloot et al., 1990; Sims et al., 1991; Wilson et al., 2011). (a) Inhibition of NCC. Predicted consequences: ↑ uptake of ions by NKCC1 → ↑ K^+^_i_ electroneutrally → ↑ electrogenic efflux of K^+^ → ↑ inside cell negativity. (b) Inhibition of NKCC1. Predicted consequences: → ↓ K^+^_i_ electroneutrally →↓  electrogenic efflux of K^+^ → ↑ inside cell positivity. (c) Inhibition of a KCC. Predicted consequences: ↑ K^+^_i_ electroneutrally → ↑ electrogenic efflux of K^+^ → ↑ inside cell negativity. Signs used in panels (a), (b), and (c) are as in legend to Figure 1. CCC, cation‐Cl^−^ cotransporter.

As mentioned already, thiazides could also play a role in bone mineralization by altering the activity of Ca^2+^‐dependent transport systems in osteoblasts given that they are known to do so in renal and intestinal epithelial cells. Based on our working model, and as illustrated through Figures 2a and 3a, they could exert part of this effect by eliciting the following series of events: ↑ K^+^ uptake by NKCC1 → ↑ K^+^_i_ → ↑ outward K^+^ conductance → ↑ inward negativity → ↑ apical Ca^2+^ uptake through conductive Ca^2+^ channels and → ↑ basolateral Ca^2+^ exit through electrogenic Na^+^/Ca^2+^ exchangers.

NKCC1 has also drawn attention in the field of BTD in that its inhibition by loop diuretics has been shown to be a risk factor for bone mass loss (Arampatzis et al., 2013; Bokrantz et al., 2020; Kubota et al., 2006; Lim et al., 2005; Norenberg, 1979; Ooms et al., 1993; Taggart, 1988). Although the mechanisms at cause are undetermined, many have incriminated the inhibitory effect of these drugs on the renal tubular reabsorption of Ca^2+^, Mg^2+^, and PO_4_^2−^ ions (Kubota et al., 2006; Rejnmark et al., 2006). More recent studies have now shown that NKCC1 could affect bone turnover because of its presence in osteoblasts. However, its subcellular localization in bone cells does not appear to have been determined as of now.

A role for NKCC1 in the skeleton was demonstrated most convincingly by Lee et al. (2003) who showed that vitamin D‐treated cultured osteoblasts responded to bumetanide by exhibiting lower levels of RANKL expression as well as JNK phosphorylation and by preventing cocultured osteoclasts to mature efficiently. These observations are further evidence for the involvement of ion transport systems in cell signaling as both osteoblastogenesis and osteoclastogenesis were seen to be affected by loop diuretics, that is, by presumed changes in the intracellular concentrations of Na^+^, K^+^, and/or Cl.^−^

Loop diuretics are not predicted to affect the electrogenic ion transport systems of osteoblasts in the same way as thiazides given that they do not act on the K^+^‐independent CCC. According once again to our model, inhibition of NKCC1 could then lead to the following succession of events: ↓ uptake of K^+^ → ↓ K^+^_i_ →↓ outward K^+^ conductance, → ↓ inward negativity → ↓ apical Ca^2+^ uptake and basolateral Ca^2+^ exit (Figures 2b and 3b). Compared to the effects of thiazides, those of loop diuretics on osteoformation should not be the same either. The study of Dvorak et al. (2007) was in fact consistent with this prediction in showing that nodule formation decreased in the presence of bumetanide.^3^

As for the KCCs, RT‐PCR studies by Brauer et al. (2003) have shown that all members of this clade were present in a human osteoblast line. Yet, the subcellular distribution of either carrier in bone forming cells has still not been reported. While inhibition of these carriers would presumably exert a thiazide‐like effect on K^+^ conductance in osteoblasts and cause osteoformation to increase, it would also bring about the added benefit of stimulating H^+^/2Cl^−^ exchange on the serosal side (see Figures 2c and 3c).

The physiological relevance of cation‐Cl^−^ cotransport in osteoblasts has not been assessed either through the skeletal characterization of bone‐specific conditional CCC mouse models. It would be most convincingly established by examining osteoformation while the activity of either isoform (that of NCC, NKCC1, KCC1, KCC3, or KCC4 in particular) is ablated or overexpressed in bone forming cells and while Ca^2+^, Mg^2+^, and PO_4_^2−^ homeostasis is kept under strict balance.

### 3.3 Osteocytes

Based on the studies available, the surface of osteocytes appears to harbor a variety of high‐conductance K^+^ channels and ion transport systems that are not present in osteoblasts (Gu et al., 2001; Ravesloot et al., 1990). Among the CCC family members, NCC is the only one to have been detected in this cell type but has been the object of no characterizations at this location (Dvorak et al., 2007). Osteocytes reside in a poorly accessible lacunocanalicular network such that their in vivo electrochemical properties are not easily amenable to light.

### 3.4 Mitochondria of bones cells

Several lines of evidence suggest that the mitochondria of osteoblasts, osteoclasts, and osteocytes play an active role in bone remodeling. In particular, mitochondrial diseases have been found to cause impaired osteogenesis and accelerated age‐related bone loss (Dobson et al., 2020). Given that the ion transport systems of mitochondria sustain oxidative phosphorylation, cell death coordination, and other key operations in these organelles, they should thus be seen as additional targets of interest in the treatment of BTD.

## 4 SUMMARY PICTURE BASED ON PERSPECTIVE PRESENTED

Based on the evidence discussed, we propose that NCC inhibition in osteoblasts increases osteoformation by allowing these cells to differentiate and express robust levels of basolateral‐to‐apical Ca^2+^/PO_4_^2−^ transport activity. In this regard, observational studies have shown that thiazides protect against bone fractures. Whether a decrease in Na^+^‐Cl^−^ cotransport at the surface osteoblasts could affect the function of osteoclasts secondarily has not been determined.

Based again on the evidence presented, NKCC1 inactivation in osteoblasts should exert the opposite effect on bone formation by preventing these cells from expressing substantial basolateral‐to‐apical Ca^2+^/PO_4_^2−^ transport activity and that it also decreases bone resorption by preventing them from secreting active pro‐osteoclastic factors. Under this premise, loop diuretics could act as a risk factor by acting directly on NKCC1 in the skeleton.

As for KCCs, their inhibition in bone cells could potentially offer important therapeutic benefits. In osteoblasts, it would presumably exert the same effect as thiazides on the vectorial flux of Ca^2+^/PO_4_^2−^ and sustain osteoid alkalinisation, and in osteoclasts, it would prevent pit acidification by decreasing H^+^ and Cl^−^ secretion. KCC inhibition could then correspond to a bone mass preserving strategy that acts on two fronts.

The WNK/OSR1‐SPAK pathway is known to inhibit the KCCs and stimulate the Na^+^‐dependent CCCs. It could thus also correspond to a pleiotropic target through which the NKCC‐to‐KCC activity ratio could be potentially increased toward therapeutic benefits. To this effect, interestingly, WNK1 expression has been found to be downregulated in the skeleton of postmenopausal women with low bone mass density (P. Xiao et al., 2008).

## 5 LIMITATIONS IN REGARD TO THE MODELS PROPOSED

Renal tubulocytes and intestinal epitheliocytes are both endowed with Ca^2+^ and PO_4_^2−^ transport mechanisms that are CCC‐sensitive. For these reasons, drugs such as thiazides or loop diuretics have been said to exert part if not all of their effects on bone density or mass by affecting whole body Ca^2+^ or PO_4_^2−^ homeostasis. Although valid, this claim is experimentally unfounded as bone‐specific loss‐of‐function or gain‐of‐function CCC mouse models do not appear to have been characterized.

A limitation of the transport schemes proposed is the paucity of data regarding the distribution of the various CCC isoforms in either of osteoblasts, osteoclasts or osteocytes. At the same time, the orientation of Ca^2+^, PO_4_^2−^, H^+^, and Cl^−^ movement by the CCC‐dependent ion transport systems would not be expected to vary as a function of where cation‐Cl^−^ movement takes place at the surface of bone cells. Be that as it may, the availability of refined localization data would certainly call for more precise transport models.

Even if there is evidence to suggest that K^+^ channel activity in bone cells is an important determinant of membrane conductance and that the movement of Na^+^, H^+^, and Cl^−^ is opposed that of K^+^ (Chow et al., 1984; Hirukawa et al., 2008), another limitation of the transport schemes proposed is that the general electrochemical properties of bone cells are poorly defined. In addition, the Na^+^/K^+^‐ATPase is known to be an important determinant of membrane conductance in many cell types, K^+^ channel activity, to vary during bone turnover and a number of K^+^ channels subtypes, to undergo rectification.

## 6 CONCLUSIONS

The CCCs could very well play crucial roles in bone turnover through their presence in osteoblasts and osteoclasts by coordinating the vectorial movement of H^+^ and Cl^−^ in one direction and that of Ca^2+^ and PO_4_^2−^ in the other. The CCCs should thus be seen as targets of interest in the treatment of BTDs, all the more so that they should be amenable eventually to bone‐targeted inhibition or perhaps even activation through relevant molecular preys and isoform‐specific drugs (Chew et al., 2019; Garneau & Isenring, 2019; Ishizaki et al., 2009; S. Liu et al., 2019).

During the last years, many fields of research appear to have been driven by the immense enthusiasm that the involvement of signaling pathways in disease development has built. Yet, these pathways play secondary or indirect pathophysiological roles in many instances and are unlikely to be completely silenced through the inhibition of a single intermediate. A change in focus might be a prelude to the identification of novel therapies that are both very safe and unexpectedly effective.

## AUTHOR CONTRIBUTIONS

*Conception, design, and drafting*: Alexandre P. Garneau, Samira Slimani, Ludwig Haydock, Fabrice Mac‐Way, and Paul Isenring. *Data acquisition*, *analysis, and interpretation*: Alexandre P. Garneau, Samira Slimani, Ludwig Haydock, Laurence E. Tremblay, Fabrice Mac‐Way, and Paul Isenring. *Critical revising*: all authors. *Table of content (figure and brief abstract)*: Thy‐René Nsimba‐Batomene, Florence C.M. Préfontaine, Mathilde M. Lavoie, and Paul Isenring. *Citation management*: all authors, especially Thy‐René Nsimba‐Batomene, Florence C.M. Préfontaine, and Mathilde M. Lavoie. All authors qualify for authorship and have approved the final version of the manuscript.

## CONFLICT OF INTEREST

The authors declare no conflict of interest.

## ENDNOTES

1 In the reminder of the text, “i” in subscript will refer to intracellular concentration and “o” in superscript to extracellular concentration.

2 The endocytic trafficking machinery is also involved in the recycling of Ca^2+^ and PO_4_^2−^ ions by osteoblasts.

3 See Fig. 4b right panel in the manuscript cited, that is, in Dvorak et al. (2007).

4 Although the localization of PIT2 in bone cells is uncertain, this transporter has been shown to be an important determinant of both bone quality and strength (Beck‐Cormier et al., 2019).

5 Surprisingly, proteomic studies have failed to identify PIT1 and PIT2 in matrix vesicles thus far (Balcerzak et al., 2008; Z. Xiao et al., 2007). NPT3/SLC17A2 is another candidate PO_4_^2−^ transport system given that it is present in bone. Several other annexins have also been identified.
